# Caregivers’ Perceptions of End-of-Life Care in Brazil: A Qualitative Systematic Review

**DOI:** 10.1590/0034-7167-2025-0220

**Published:** 2026-08-03

**Authors:** Fernanda Cirne Lima Weston, Aline Branco, Isabela Ouriques Borghetti, Irani Iracema de Lima Argimon

**Affiliations:** I Pontifícia Universidade Católica do Rio Grande do Sul. Porto Alegre, Rio Grande do Sul, Brazil; II Grupo Hospitalar Conceição. Porto Alegre, Rio Grande do Sul, Brazil

**Keywords:** Palliative Care, Terminal Care, End of Life Care, Caregivers, Caregiver Burden, Death., Cuidados Paliativos, Cuidado Terminal, Cuidado en el Final de la Vida, Cuidadores, Carga del Cuidador, Muerte.

## Abstract

**Objectives:**

to identify barriers and facilitators perceived by informal caregivers that impact care for adults and older adults at the end of life in Brazil.

**Methods:**

a systematic review of qualitative studies, following the JBI model, with meta-aggregation analysis.

**Results:**

thirteen studies were included. Five synthesized findings were identified. Caregivers reported the following as positive aspects of end-of-life care: access to a skilled and caring multidisciplinary team, the presence of an informal and, especially, family support network, clear communication addressing end-of-life issues, the development of resilience, and support in beliefs and religions. Barriers identified included a lack of preparedness among professionals and in hospital settings to care for this population, and feelings of physical, psychological, and financial overload.

**Final Considerations:**

the findings have practical implications, allowing for the development of strategies to mitigate the difficulties faced by caregivers in the Brazilian context.

## RESUMEN

**Objetivos:**

identificar las barreras y facilitadores percibidos por los cuidadores informales que impactan en el cuidado de adultos y ancianos al final de la vida en Brasil.

**Métodos:**

revisión sistemática de estudios cualitativos, siguiendo el modelo del JBI, con análisis de metaagregación.

**Resultados:**

se incluyeron trece estudios. Se identificaron cinco hallazgos sintetizados. Los cuidadores informaron los siguientes aspectos positivos de los cuidados al final de la vida: el acceso a un equipo multidisciplinario capacitado y atento, la presencia de una red de apoyo informal y, especialmente, familiar, una comunicación clara para abordar cuestiones relacionadas con el final de la vida, el desarrollo de la resiliencia y el apoyo en creencias y religiones. Las barreras identificadas incluyeron la falta de preparación de los profesionales y del entorno hospitalario para atender a esta población, así como la sensación de sobrecarga física, psicológica y financiera.

**Consideraciones Finales:**

los resultados tienen implicaciones prácticas, lo que permite el desarrollo de estrategias para mitigar las dificultades que enfrentan los cuidadores en el contexto brasileño.

## INTRODUCTION

The Brazilian population grew by approximately 7.6% between 2012 and 2021, and the number of older adults showed a higher growth rate than all other age groups during the period studied^(1)^. This demographic shift, characterized by an increase in life expectancy, results in a higher prevalence of chronic non-communicable diseases, which require a structured healthcare service to address them^(2)^.

Within this category, adult and older patients at the end of life, i.e., those with a life expectancy of months or less, require constant care, as they experience an intensification of symptoms such as pain and fatigue^(3-5)^. These patients are eligible for palliative care, although these services are not exclusive to people at the end of life, and may also exhibit reduced levels of consciousness, difficulty walking, and other barriers to self-care, making the presence of an informal caregiver essential^(5)^.

Informal caregivers typically lack professional qualifications, providing care based on relational principles. Therefore, they include family members, friends, or other individuals who do not receive remuneration for their caregiving. Motivations for providing care include cultural values and social norms, ranging from religious beliefs, such as the idea that a person is placed in a caregiver role by God’s will, to the perception that the healthcare system is insufficient, necessitating the constant presence of a family member to prevent errors^(6)^.

Informal caregivers take on tasks such as feeding patients, administering medication, and providing accompaniment during hospitalizations, offering emotional support^(7)^. These activities, in addition to representing a significant physical and emotional burden, make them a constant and daily presence in patients’ lives, allowing them to identify the barriers and facilitators for the comprehensive care of a person at the end of life. “Barrier” is defined as factors that prevent and/or hinder the physical, psychological, social, and spiritual needs of a person at the end of life from being met. Conversely, “facilitating” factors are those that allow or encourage the fulfillment of a patient’s comprehensive needs^(8)^.

International research demonstrates that there are multiple barriers and facilitators that impact the use of palliative care services. Lack of knowledge and fear of palliative care, both on the part of professionals and caregivers and patients, difficulty of access in rural areas, and lack of communication between physicians and the multidisciplinary team are examples of factors that hinder access to these services, and can also be identified by informal caregivers of a person at the end of life^(9,10)^.

Obstacles to end-of-life patient care vary between countries due to different healthcare service structures, culture, and the predominant religion of the population^(10)^. Therefore, this review is constructed focusing on studies developed nationally in order to respect these peculiarities.

A preliminary search was conducted in the PROSPERO and LILACS databases in December 2023 to verify if there was a systematic review that sought to map the barriers and facilitators identified by informal caregivers in the care of end-of-life patients in Brazil. However, despite the existence of Brazilian research addressing caregivers’ perceptions and experiences, no reviews were found that sought to gather and systematize them^(7,11)^. Considering that these caregivers’ perceptions can be used as a resource to improve end-of-life care and healthcare services, it becomes necessary to understand and systematize them.

## OBJECTIVES

To identify the barriers and facilitators perceived and experienced by informal caregivers in the care of adults and older adults at the end of life in Brazil.

## METHODS

### Ethical aspects

Since this was a systematic review, approval from the Research Ethics Committee was not required.

### Research design

A systematic review of qualitative studies was chosen, following the methodology proposed by JBI^(12)^. Qualitative studies allow, through individuals’ perspective, the identification of weaknesses and strengths of health interventions, understanding why they are applicable or not in each context. The study protocol was registered (CRD42024523211) on the PROSPERO platform.

### Research question identification

The research question, “What are the perceived and experienced barriers and facilitators for informal caregivers in the care of adult and older patients at the end of life in Brazil?”, was formulated using the acronym PICo (Population, Phenomenon of Interest, and Context): P - informal caregivers of patients; I - barriers and facilitators in healthcare; and Co - end-of-life care^(12)^. As inclusion criteria, the selected materials should be primary qualitative studies, including approaches such as case studies and ethnography, conducted in Brazil, available online in full, and focusing on the perceptions of informal caregivers who provide or have provided assistance to a patient at the end of life. Articles that also addressed healthcare professionals’ perceptions could be included, provided it was possible to identify whether interviewees were informal or formal caregivers, allowing only the statements of informal caregivers to be analyzed for this study.

Studies that addressed caregivers of pediatric patients (<18 years) and materials that did not explicitly state patients’ life expectancy or define them as end-of-life patients were excluded. Studies that used only terms such as “negative prognosis” and “in palliative care”, without specifying life expectancy or defining patients as end-of-life and/or terminally ill (used in this article as synonyms^(3)^), were not included in selection. In the case of both articles derived from theses/dissertations and original theses/dissertations being found, the published articles were selected for review.

### Search strategy

The search was conducted on March 15, 2024, covering published and grey literature databases^(12)^, as recommended by JBI. Database searches were conducted using Medical Subject Headings descriptors, and the search strategy was developed with the support of a librarian specializing in the health field (Chart 1). Descriptors were applied to the Scopus, PubMed, LILACS, SciELO, and Web of Science databases, and to locate grey literature, the SciELO Preprints and the Coordination for the Improvement of Higher Education Personnel (In Portuguese, *Coordenação de Aperfeiçoamento de Pessoal de Nível Superior* - CAPES) Catalog of Theses and Dissertations databases were also used.

**Chart 1:** Search strategies used in databases, Porto Alegre, Rio Grande do Sul, Brazil, 2024

Source of information	Search strategy
LILACS	(“Terminally Ill” OR “Ill, Terminally” OR “end of life care” OR “End of Life Care” OR “End-Of-Life Care” OR “Care, End-Of-Life” OR “End-Of-Life Cares” OR “Terminal Care” OR “Care, Terminal”) AND (caregivers OR caregiver OR carers OR carer OR “Care Givers” OR “Care Giver” OR “Spouse Caregivers” OR “Caregiver, Spouse” OR “Caregivers, Spouse” OR “Spouse Caregiver” OR “Family Caregivers” OR “Caregiver, Family” OR “Caregivers, Family” OR “Family Caregiver” OR “Informal Caregivers” OR “Caregiver, Informal” OR “Caregivers, Informal” OR “Informal Caregiver”) AND ( db:(“LILACS”))
PubMed	(“Terminally Ill”[MeSH Terms] OR “ill terminally”[All Fields] OR “Terminal Care”[MeSH Terms] OR “end of life care”[All Fields] OR “end of life care”[All Fields] OR “end of life care”[All Fields] OR “care end of life”[All Fields] OR “End-Of-Life Cares”[All Fields] OR “care terminal”[All Fields]) AND (“caregivers”[MeSH Terms] OR (“caregiver s”[All Fields] OR “caregivers”[MeSH Terms] OR “caregivers”[All Fields] OR “caregiver”[All Fields] OR “caregiving”[All Fields]) OR (“caregivers”[MeSH Terms] OR “caregivers”[All Fields] OR “carer”[All Fields] OR “carers”[All Fields] OR “carer s”[All Fields]) OR (“caregivers”[MeSH Terms] OR “caregivers”[All Fields] OR “carer”[All Fields] OR “carers”[All Fields] OR “carer s”[All Fields]) OR “Care Givers”[All Fields] OR “Care Giver”[All Fields] OR “Spouse Caregivers”[All Fields] OR “caregiver spouse”[All Fields] OR “caregivers spouse”[All Fields] OR “Spouse Caregiver”[All Fields] OR “Family Caregivers”[All Fields] OR “caregiver family”[All Fields] OR “caregivers family”[All Fields] OR “Family Caregiver”[All Fields] OR “Informal Caregivers”[All Fields] OR “caregiver informal”[All Fields] OR “caregivers informal”[All Fields] OR “Informal Caregiver”[All Fields]) AND (“brasil”[All Fields] OR (“brazil”[MeSH Terms] OR “brazil”[All Fields] OR “brazil s”[All Fields] OR “brazils”[All Fields]) OR (“brazilian people”[Supplementary Concept] OR “brazilian people”[All Fields] OR “brazilians”[All Fields] OR “brazilian”[All Fields]) OR (“brazilian people”[Supplementary Concept] OR “brazilian people”[All Fields] OR “brazilians”[All Fields] OR “brazilian”[All Fields]))
Scopus	TITLE-ABS-KEY ( ( “Terminally Ill” OR “Ill, Terminally” OR “end of life care” OR “End of Life Care” OR “End-Of-Life Care” OR “Care, End-Of-Life” OR “End-Of-Life Cares” OR “Terminal Care” OR “Care, Terminal” ) AND ( caregivers OR caregiver OR carers OR carer OR “Care Givers” OR “Care Giver” OR “Spouse Caregivers” OR “Caregiver, Spouse” OR “Caregivers, Spouse” OR “Spouse Caregiver” OR “Family Caregivers” OR “Caregiver, Family” OR “Caregivers, Family” OR “Family Caregiver” OR “Informal Caregivers” OR “Caregiver, Informal” OR “Caregivers, Informal” OR “Informal Caregiver” ) AND ( brasil OR brazil OR brazilian or brazilians ) )
Web of Science	(“Terminally Ill” OR “Ill, Terminally” OR “end of life care” OR “End of Life Care” OR “End-Of-Life Care” OR “Care, End-Of-Life” OR “End-Of-Life Cares” OR “Terminal Care” OR “Care, Terminal”) AND (Caregivers OR Caregiver OR Carers OR Carer OR “Care Givers” OR “Care Giver” OR “Spouse Caregivers” OR “Caregiver, Spouse” OR “Caregivers, Spouse” OR “Spouse Caregiver” OR “Family Caregivers” OR “Caregiver, Family” OR “Caregivers, Family” OR “Family Caregiver” OR “Informal Caregivers” OR “Caregiver, Informal” OR “Caregivers, Informal” OR “Informal Caregiver”) AND (Brasil OR Brazil OR brazilian OR brazilians) (All Fields)
SciELO Preprints	(“Terminally Ill” OR “Ill, Terminally” OR “end of life care” OR “End of Life Care” OR “End-Of-Life Care” OR “Care, End-Of-Life” OR “End-Of-Life Cares” OR “Terminal Care” OR “Care, Terminal”) AND (Caregivers OR Caregiver OR Carers OR Carer OR “Care Givers” OR “Care Giver” OR “Spouse Caregivers” OR “Caregiver, Spouse” OR “Caregivers, Spouse” OR “Spouse Caregiver” OR “Family Caregivers” OR “Caregiver, Family” OR “Caregivers, Family” OR “Family Caregiver” OR “Informal Caregivers” OR “Caregiver, Informal” OR “Caregivers, Informal” OR “Informal Caregiver”)
SciELO (Portuguese)	*(“Doente Terminal”* OR *“Paciente Terminal”* OR *“Assistência Terminal”* OR *“Cuidados de Fim de Vida”)* AND *(Cuidador* OR *“Cuidador Familiar”* OR *“Cuidador de Família”* OR *“Cuidadores Cônjuges”* OR *“Cuidadores Familiares”* OR *“Cuidadores Informais”* OR *“Cuidadores de Família” OR “Cônjuges Cuidadores”* OR *“Familiar Cuidador”* OR *“Familiares Cuidadores”* OR *“Outro Apoiador”)*
SciELO (English)	(“Terminally Ill” OR “Ill, Terminally” OR “end of life care” OR “End of Life Care” OR “End-Of-Life Care” OR “Care, End-Of-Life” OR “End-Of-Life Cares” OR “Terminal Care” OR “Care, Terminal”) AND (caregivers OR caregiver OR carers OR carer OR “Care Givers” OR “Care Giver” OR “Spouse Caregivers” OR “Caregiver, Spouse” OR “Caregivers, Spouse” OR “Spouse Caregiver” OR “Family Caregivers” OR “Caregiver, Family” OR “Caregivers, Family” OR “Family Caregiver” OR “Informal Caregivers” OR “Caregiver, Informal” OR “Caregivers, Informal” OR “Informal Caregiver”)
Coordination for the Improvement of Higher Education Personnel Catalog of Theses and Dissertations	*Terminal* AND *Cuidadores*

An initial search was conducted in the PubMed database with the assistance of a librarian to verify if the strategy was appropriate for the proposed research question. After verifying the initial results, the search strategy was applied to all databases. The searches were performed through the institutional access of the CAPES Federated Academic Community. No language filters were applied, nor was there a restriction on the year of publication.

### Data analysis

The materials found in the databases were included in the Rayyan^®^ application for better organization and duplicate detection. Study screening is presented using the Preferred Reporting Items for Systematic Reviews and Meta-Analyses (PRISMA) criteria^(13)^. The search process was conducted by two researchers, who began by reading the titles and abstracts of the returned articles and subsequently read the articles in full. During study screening, a third researcher was involved in cases of discrepancies in the decisions that could not be resolved after discussion between the two initial researchers.

After screening the studies using Rayyan^®^, the articles were entered into the JBI SUMARI^®^ software for application of the Checklist for Qualitative Research critical appraisal instrument, completed by two researchers for subsequent comparison of the assessments. Only studies that, in addition to meeting the inclusion criteria already mentioned, fulfilled criteria 2, 4, 8, and 10 of the checklist were selected for review.

Data mapping was performed using the JBI Qualitative Data Extraction Tool, an instrument completed independently by two researchers, whose results were subsequently compared. Data analysis was conducted using meta-aggregation, as it allows for the identification of patterns across different interviews and also directs the results towards clinical practice^(12)^.

Following meta-aggregation, the first step in analyzing the studies consisted of reading them in full to identify the findings, which are literal extractions from the author’s words. All findings must be accompanied by an illustration, which is a literal transcription of interviewees’ speeches. After combining the findings with illustrations, the researchers were responsible for assigning a level of credibility to the combination. This is divided into unequivocal (where an illustration is clearly associated with the findings), credible (where an illustration has an association with the findings, but not as clear), and unsupported (where an illustration does not support the author’s interpretation). All unsupported findings were not considered for the analysis of results.

After assigning a level of credibility, the second stage of meta-aggregation was undertaken, creating categories from the grouping of findings that had similar meaning. Finally, the categories that shared similar meanings were also grouped into so-called synthesized findings. The synthesized findings were considered highly reliable when composed only of unequivocal categories; moderately reliable when composed of unequivocal and credible categories; and of low reliability when composed only of credible categories.

## RESULTS

**Figure 1:**
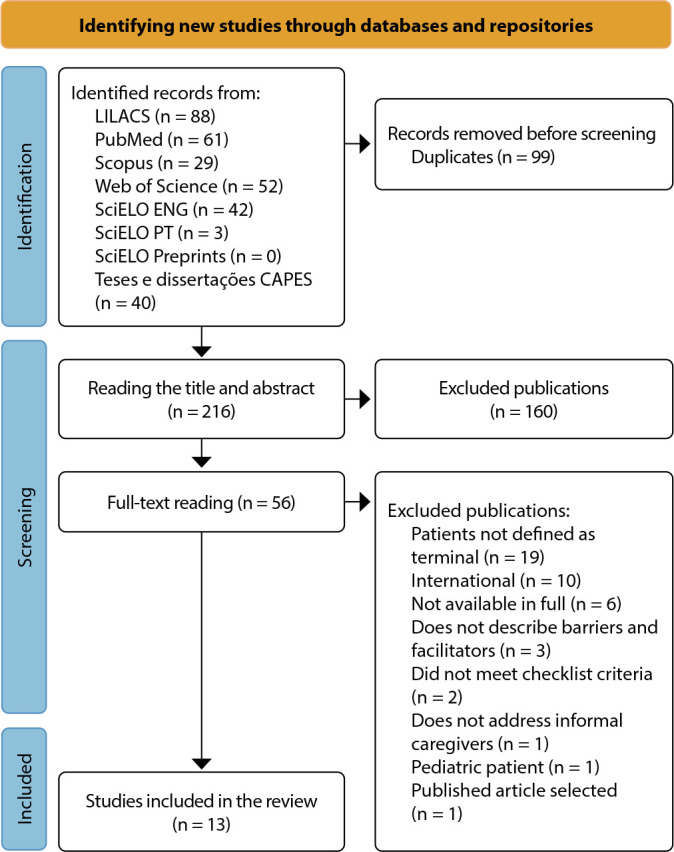
Preferred Reporting Items for Systematic Reviews and Meta-Analyses Flowchart, Porto Alegre, Rio Grande do Sul, Brazil, 2024

After applying the search strategies to the databases, a total of 315 publications were identified, as per the PRISMA flowchart (Figure 1). After removing duplicates, 216 remained. After reading the title and abstract, 160 were excluded, and only in one conflict was a third researcher consulted. Moreover, 56 materials were selected for full-text reading. Of these 56, 15 were selected by reading and, after applying the Checklist for Qualitative Research, 13 were selected for a systematic review.

Of the materials selected, nine (69.2%) were carried out in home hospitalization services^(14-22)^ and five (38.5%) in a hospital setting^(7,11,21,23,24)^. One of the articles was conducted in both modalities^(20)^. Among all the studies, eight (61.5%) were conducted in the South region, three (23%) in the Southeast region, and two (15.4%) in the Northeast region (Chart 2).

**Chart 2:** Characteristics of selected studies, Porto Alegre, Rio Grande do Sul, Brazil, 2024

Authors (Year)	Sample | Patient diagnosis	Local (region)	Data collection instrument
Oliveira *et al.* (2011)^(14)^	11 family caregivers | Cancer	Home hospitalization service linked to a university hospital (South)	Narrative interviews
Oliveira *et al.* (2012)^(15)^	11 family caregivers | Cancer	Home hospitalization service linked to a university hospital (South)	Narrative interviews
Vasques *et al.* (2017)^(23)^	24 family caregivers | Not identified	Medical clinic unit (South)	Observation and interview
Oliveira *et al.* (2013)^(16)^	11 family caregivers | Cancer	Home hospitalization service linked to a university hospital (South)	Narrative interviews
Barbosa *et al.* (2020)^(7)^	15 family caregivers | Cancer	Philanthropic hospital (Northeast)	Semi-structured interviews
Nietsche *et al.* (2013)^(17)^	Two nurses, one nursing assistant, one physician, one nutritionist, and four family caregivers | Not identified	Home hospitalization service linked to a university hospital (South)	Semi-structured interviews
Cogo *et al.* (2016)^(18)^	Eight nurses, seven physicians, and seven family caregivers | Not identified	Home hospitalization service linked to a university hospital (South)	Semi-structured interview
Oliveira *et al.* (2012) ^(19)^	11 family caregivers | Cancer	Home hospitalization service linked to a university hospital (South)	Narrative interviews
Souza & Turrini (2011)^(24)^	Seven family caregivers | Cancer	Outpatient service of a public university hospital (Southeast)	Non-directive interviews
Santos (2019)^(21)^	18 family caregivers | Cancer	Home care service linked to a palliative care unit of a philanthropic hospital (Northeast)	Open interview
Benites AC *et al.* (2022)^(11)^	Ten family caregivers | Cancer	Hospital and cancer center (Southeast)	Phenomenological interview
Santos MJ (2009)^(20)^	Ten family caregivers, three nurses, four nursing technicians, and one nursing assistant | Cancer	Home-based, outpatient and inpatient palliative care services (South)	Semi-structured interview
Fonseca COS (2012)^(22)^	Seven family caregivers | Cancer	Home hospitalization service (Southeast)	Semi-structured interview

Concerning the diagnoses studied, ten (76.9%) studies addressed caregivers of cancer patients, although they did not specify the type, and three (23.1%) did not identify the diagnosis. After performing meta-aggregation, five synthesized findings were identified, presented below along with the categories that compose them (Chart 3).

**Chart 3:** Synthesized findings and categories identified through meta-aggregation, Porto Alegre, Rio Grande do Sul, Brazil, 2024

Synthesized findings	Categories
1. Caregivers report that easy access to a skilled and caring multidisciplinary team that respects the autonomy of end-of-life patients is central to providing good care	Access to a multidisciplinary team
	Caring and respectful multidisciplinary team
	Shared care and tasks with professionals
	Nursing team support
	Home care
	Difficulty in identifying and meeting patients’ needs
2. Caregivers state that having an informal support network is essential for sharing caregiving responsibilities, thus avoiding physical, psychological, and financial overload	Family support
	Physical and emotional overload
	Lack of family support
	Loneliness
	Financial difficulties
	Creating a support network
3. Caregivers recognize the importance of clear and effective communication addressing the topic of death and dying among the multidisciplinary team, the caregiver, and the patient	Inadequate communication between the caregiver, the patient, and the multidisciplinary team
	Communication that allows for discussion about death
	Adequate communication with and among the multidisciplinary team
4. Caregivers perceive a lack of preparedness of professionals and hospital settings for caring for end-of-life patients, and they also distrust palliative care services	Disengagement from the multidisciplinary team
	Limitations of advance directives
	Hospital setting
	Distrust of palliative care and other healthcare services
5. Caregivers describe different tools, such as developing resilience and relying on beliefs and religions, for accepting terminal illness and coping with the caregiver role	Resilience
	Beliefs and religion
	Acceptance of death

### Synthesized finding 1: Caregivers report that easy access to a skilled and caring multidisciplinary team that respects the autonomy of end-of-life patients is central to providing good care

This synthesized finding was created from six categories and is considered moderately reliable, encompassing 24 unequivocal findings and four credible findings. According to caregivers’ perceptions, the preference for a care setting, whether home or hospital, is primarily due to easy access to a multidisciplinary team.

The category called “Access to a multidisciplinary team” consists of four findings. Some caregivers report that they prefer hospital settings because of the constant presence of healthcare professionals. Regarding home care services, they feel safe having the presence and telephone contact of the team and knowing that they can contact them 24 hours a day for questions and support. They report that already knowing some of the home care service members makes them less concerned about accessing it.

The “Caring and respectful multidisciplinary team” category consists of eight findings, highlighting the importance of having access to a multidisciplinary team that demonstrates patience, affection, respect, and availability to both patients and caregivers. Active listening and respect for patients’ needs and desires are valued by caregivers.

Regarding the “Shared care and tasks with professionals” category, six findings comprise it, with caregivers feeling safer and less burdened when care and daily tasks are shared with a professional, who can be either a healthcare team member or a domestic worker responsible for cleaning and organizing the house. They cite the guidance received from the home care service’s healthcare team, as well as division of tasks, such as a rotation in performing wound care between formal and informal caregivers.

The “Nursing team support” category comprises three findings. Caregivers value the support and comfort specifically provided by the nursing team, perceiving them as supportive both at home and in hospital settings. Meanwhile, the “Home care” category comprises three findings. Caregivers perceive that home care services facilitate access to hospital resources, such as medical equipment and emergency care. They also recognize that this modality allows for greater autonomy on the part of the patient.

Finally, the “Difficulty in identifying and meeting patients’ needs” category consists of four findings, demonstrating the difficulties reported by caregivers in identifying and managing patients’ health needs. Issues such as uncontrolled pain and increased patient irritability impact caregivers’ perception of their capabilities, making them apprehensive about not providing adequate care.

### Synthesized finding 2: Caregivers state that having an informal support network is essential for sharing caregiving responsibilities, thus avoiding physical, psychological, and financial overload

Created from six categories, this synthesized finding is considered moderately reliable, encompassing 28 findings, two of which are moderate and 26 unequivocal. It emphasizes the importance of a community and, especially, family support network, both in sharing care and in providing financial support. The possibility of intra-family care rotation would reduce the feeling of overload and loneliness of the primary caregiver who sometimes fears being solely responsible should the patient die.

The “Family support” category comprises three findings, highlighting the importance of caregivers having access to a family support network for rotating care of end-of-life patients. It cites a preference for home care when there is help from other family members, and values the emotional support they receive from family members. Meanwhile, the “Physical and emotional overload” category consists of ten findings. There are different feelings and physical symptoms that emerge from caring for end-of-life patients and hinder their ability to cope, including fear, anxiety, anguish, depression, fatigue, stress, despair, discouragement, and lack of recognition. It is noteworthy that being a mother caring for a child at the end of life further complicates the grieving process, increasing the emotional burden.

The “Lack of family support” category, comprised of five findings, demonstrates the experiences of caregivers when there is a lack of family support, including lack of financial support and conflicts between family members. A gender bias is evident in these experiences, and caregivers are usually women who do not receive support from other male family members. There is a fear of being served by home care services, as caregivers may be held responsible by other family members if a patient dies at home.

Meanwhile, the “Loneliness” category is composed of three findings. It refers to caregivers’ feeling of being alone and isolated in caring for end-of-life patients, including upon receiving the initial diagnosis of the disease. The “Financial difficulties” category, comprised of five findings, demonstrates the financial limitations that impact the care, access, and transportation to healthcare services for end-of-life patients. Caregivers report conflict with their employers both during hospitalizations and home care, due to the time spent with patients, culminating in their dismissal.

Finally, the “Creating a support network” category is composed of two findings. It highlights the importance caregivers place on dialogue and the bond established with other patients and caregivers who are also hospitalized in similar situations.

### Synthesized finding 3: Caregivers recognize the importance of clear and effective communication addressing the topic of death and dying among the multidisciplinary team, the caregiver, and the patient

This synthesized finding is composed of three categories and has high reliability, consisting of eight unequivocal findings. Caregivers’ reports acknowledge the importance of communication between the triad formed by patients, primary caregivers, and care team. They also affirm greater safety and confidence when they perceive integrated communication among different members of the multidisciplinary team. It notes that dialogues about death and the dying process are difficult, but necessary.

The “Inadequate communication between the caregiver, the patient, and the multidisciplinary team” category, comprised of four findings, reveals that the use of technical-scientific language and vague explanations by professionals to explain the negative prognosis negatively impacts the care of end-of-life patients. The lack of communication, both from the team and from caregivers, regarding end-of-life care is also recognized as a barrier to assistance. The “Communication that allows for discussion about death” category, consisting of two findings, demonstrates the importance of establishing communication with the multidisciplinary team and patientd that allows discussion about death, enabling the sharing of accurate information between caregiver, patient, and family members.

In contrast, the “Adequate communication with and among the multidisciplinary team” category, comprised of two findings, refers to the importance of establishing effective and clear communication among multidisciplinary team members, as well as between them and caregivers. It also highlights the need for integration within the team so that everyone conveys the same information to caregivers.

### Synthesized finding 4: Caregivers perceive a lack of preparedness of professionals and hospital settings for caring for end-of-life patients, and they also distrust palliative care services

Created from four categories, this synthesized finding addresses issues related to the detachment of the multidisciplinary team from end-of-life patients, the limitations perceived by caregivers regarding the application of advance directives (ADs), the characteristics of hospital settings, and distrust or lack of knowledge about palliative care and other healthcare services. It is considered moderately credible, consisting of two credible and eight unequivocal categories.

The “Disengagement from the multidisciplinary team” category comprises two findings, addressing caregivers’ perception that the multidisciplinary team avoids approaching end-of-life patients. Family members observe that, upon the establishment of a negative prognosis, healthcare professionals’ assistance to patients decrease. The “Limitations of advance directives” category, consisting of three findings, further exposes the lack of professional preparedness and legal support, which impact the implementation of ADs. Caregivers have concerns related to the potential legal implications of ADs and demonstrate a hope for patients’ recovery at the end of life, limiting the implementation of ADs.

Caregivers’ perceptions of the “hospital space” also demonstrate the characteristics of hospital settings that impact end-of-life patient care. They highlight the negative aspects of this space, emphasizing that it makes exercising autonomy more difficult, and there is a greater chance of patients contracting an infection, the latter mainly because the rooms are shared with other people.

Finally, the “Distrust of palliative care and other healthcare services” category comprises three findings. Informal caregivers perceive that their distrust of healthcare services and lack of knowledge about the purpose of palliative care impact access to these services. They distrust the quality of care provided by the Brazilian Health System. Regarding palliative care, they have a preconceived notion that it is a service people only access when they are dying. Caregivers also report feeling afraid to hire someone to assist in the care of end-of-life patients for fear that their family member will be mistreated.

### Synthesized finding 5: Caregivers describe different tools, such as developing resilience and relying on beliefs and religions, for accepting terminal illness and coping with the caregiver role

This synthesized finding is composed of three categories and has moderate reliability, presenting seven unequivocal categories and one credible one. It refers to the development of resilience, the role of beliefs and religion in supporting caregivers, and acceptance of death.

Regarding “Resilience”, comprised of four findings, adaptive feelings that emerge in situations of risk and adversity are presented. This includes the emergence of courage and feelings of love in caregivers, which seem to strengthen them in the face of the challenge of caregiving. It addresses the existence of feelings related to gratitude and the mechanism of humor, through “being a clown”.

The “Beliefs and religion” category, comprised of three findings, discusses beliefs and religion as a source of support for caregivers. It highlights the value of faith and religiosity in the caregiving process. Caregivers report that religion, faith, and belief in life after death seem to facilitate the process of accepting death, as well as helping them understand the suffering experienced.

Finally, the “Acceptance of death” category consists of three findings, presenting caregivers’ recognition and acceptance of patients’ terminal illness. It addresses the acceptance of the end of life and the ability to prepare beforehand for the moments following patients’ death, such as preparing funeral rites. It also addresses the acceptance of death through the view that it represents the end of suffering.

## DISCUSSION

The findings of the studies raise some reflections on the different barriers and facilitators identified by informal caregivers in providing care to end-of-life patients in Brazil. They report that easy access to a trained and caring multidisciplinary team is central to providing good care, both in home care services and in hospital settings.

It is necessary to consider this information when operationalizing end-of-life care services in Brazil, understanding the importance for caregivers of the presence and constant contact with healthcare professionals for support and guidance. The literature primarily demonstrates the relevance of offering telephone numbers that are available after business hours to address concerns regarding the worsening of common physical and psychological symptoms in the final stages of life^(25,26)^.

It is recognized that the limitation of financial and human resources, common in different regions and institutions in Brazil, can hinder or prevent the provision of these services^(24)^. However, it is important to highlight the positive impacts of having constant access to a multidisciplinary team, which, in addition to reducing patient symptoms, also reduces feelings of overload and improves caregivers’ well-being and mood^(27)^.

Beyond the importance of sharing care with a technically prepared team, caregivers also value professionals who demonstrate affection and respect for both patients and their family. Guidelines from different countries, such as Australia and Singapore, highlight the importance of the multidisciplinary team communicating honestly, sensitively, and respecting patients’ wishes and needs in order to create a bond of trust^(28)^. National guidelines in Canada and India also list empathetic and compassionate communication as a necessary competency for end-of-life care, supporting the Brazilian findings^(29,30)^.

In addition to being able to share patient care with a multidisciplinary team, caregivers also need an informal support network, mainly composed of family members. Other studies have demonstrated the importance of not assigning caregiving tasks solely to primary caregivers, but sharing them among various family members^(31)^. In this review, caregivers perceive that rotating caregiving responsibilities prevents physical and psychological overload, as well as enabling care to be provided in a home setting, rather than a hospital.

Furthermore, caregivers report the importance of receiving financial support from other family members so that they can access healthcare services and maintain proper treatment. This need is also observed in research in other developing countries: in Uganda, as in Brazil, expenses for public transport and food overburden primary caregivers, forcing them to sell assets and use all their savings^(32)^. In both nations, there are also reports of constant conflicts with employers, resulting in layoffs and even greater financial hardship.

In addition to the need for family support, caregivers recognize the importance of clear and effective communication addressing death among the multidisciplinary team, caregiver, and patient. Effective communication is understood as that which occurs clearly, directly, jointly, and routinely, and its insufficiency becomes one of the main factors contributing to adverse events and a decrease in patients’ quality of life^(33)^.

Although this is the only synthesized finding of high reliability, healthcare professionals state that they are not open to discussing end-of-life issues with patients’ families. A study conducted in Sergipe demonstrates that the greatest difficulties in implementing ADs are related to family resistance generated by a misunderstanding of patients’ true prognosis. In the same research, the team is cautious regarding family members’ emotional responses, showing a tendency to avoid feelings of stress, guilt, failure, and helplessness that may arise depending on caregivers’ reaction^(34)^.

Contrary to this finding, a study conducted in England demonstrates that terminally ill patients and their family caregivers are resistant to addressing the topic of death and dying, always bringing up the subject indirectly^(35)^. Interestingly, the author also points out that family members expect these issues to always be raised by the multidisciplinary team and that such an approach should be direct, supporting the findings of this review.

Caregivers also perceive the lack of preparedness of professionals and the hospital to care for end-of-life patients as a barrier. During hospitalization, there is a noticeable distancing of the multidisciplinary team when a negative prognosis is established, which is also identified by the healthcare professionals themselves. In different studies conducted in Brazil, nurses and nursing technicians report that co-workers distance themselves from the person receiving end-of-life care and fail to perform procedures necessary for patients’ comfort, attributing this behavior to professionals’ inability to cope with suffering and death^(36,37)^.

The proximity to death is also a challenge for informal caregivers, who report different tools for accepting terminal illness and fulfilling their role. The development of resilience, defined as the ability to adapt positively to adverse situations, is perceived as necessary for coping with death^(38)^. Good humor, gratitude, and feelings of love emerge in other studies conducted with caregivers of terminally ill and dementia patients, and are considered psychological strengths that can be encouraged and strengthened to improve caregivers’ quality of life^(39,40)^.

In addition to developing resilience, caregivers frequently seek support in beliefs and religion. According to the European Association for Palliative Care, spirituality is a dynamic dimension of human life that relates to how people connect to a particular moment in life, to themselves, to others, and to the sacred, and contributes to the development of hope for the future^(41)^. Meanwhile, religious rituals aid in the process of accepting death and loss, developing the notion of closure and facilitating informal caregivers’ self-awareness towards a greater appreciation of life events.

A study conducted in Brazil demonstrates the correlation between spirituality and the emotional burden experienced by family caregivers of patients under exclusive palliative care^(42)^. Family members with a high level of spirituality feel less overwhelmed, while caregivers with lower levels of spirituality are at greater risk of emotional overload, anxiety, and stress, opening up an area that needs to be addressed and supported by healthcare professionals.

### Study limitations

This review allows for the identification of the main barriers and facilitators faced by informal caregivers when caring for end-of-life individuals in Brazil, but its limitations must be highlighted. None of the selected studies were conducted in the North and Central-West regions, raising questions about whether results can be generalized to these contexts. The research was also predominantly conducted with caregivers of cancer patients, raising questions about the applicability of the findings to other diagnoses such as neurodegenerative and cardiac diseases, among others.

### Contributions to nursing

Despite the limitations, the results found have practical implications and allow for the development of strategies to mitigate the difficulties faced by caregivers in the national context. Institutional approaches such as maintaining a 24-hour telephone line for questions from informal caregivers and strengthening an organizational culture that values healthcare professionals’ mental health can positively impact the care of individuals at the end of life. Training for healthcare professionals, especially regarding how to address the topic of death in their communication with patients and caregivers, could also be considered beneficial.

Promoting resilience and respecting spirituality, as well as reinforcing the importance of rotating family tasks from the beginning of treatment, can impact caregivers’ quality of life, consequently improving patient care. Therefore, nursing consultations with caregivers and end-of-life patients should explicitly guide informal caregivers to organize an informal support network, aiming to share caregiving and reduce physical, psychological, and financial burden.

Further studies can be conducted to assess the effectiveness and applicability of these interventions, including in resource-limited settings, which are common in the Brazilian context. A gap in knowledge is also identified regarding why healthcare professionals distance themselves from the topic of death. Research is needed to understand this phenomenon, with the aim of preventing it and making care more emotionally sustainable for formal caregivers.

## FINAL CONSIDERATIONS

Various facilitators and barriers are reported by informal caregivers in providing care to people at the end of life in Brazil. Access to a trained and caring multidisciplinary team, the presence of an informal and, especially, family support network, clear and effective communication addressing the topic of end-of-life care, the development of resilience, and support in beliefs and religions are factors that caregivers perceive as positive aspects of caring for people at the end of life.

On the other hand, they observe barriers such as the lack of preparedness of professionals and hospital settings to care for this population. They also report feelings of physical, psychological, and financial overload, associating them mainly with the lack of family support received.

Analyzing these barriers and facilitators allows healthcare professionals to develop support strategies better adapted to these caregivers’ experiences. For instance, during a nursing consultation, a valuable practice would be to support and guide the caregiver in identifying their formal and informal support network in order to avoid overload. Professional training in communicating bad news is also fundamental for an effective approach to end-of-life care. However, it is recognized that improving these conditions will require not only professional training, but also effective public policies and institutional reorganizations that acknowledge and mitigate the burden faced by informal caregivers in Brazil.

## Data Availability

The research data are available within the article.
