# Supplementary material for: Caregivers’ Perceptions of End-of-Life Care in Brazil: A Qualitative Systematic Review
Source: Rev Bras Enferm. 2026 Aug 3;79:e20250220. doi: 10.1590/0034-7167-2025-0220 (PMC13432807; doi:10.1590/0034-7167-2025-0220)
Supplement: Translation

# Translation: Percepções dos Cuidadores sobre os Cuidados de Fim de Vida no Brasil: Revisão Sistemática Qualitativa

**DOI:** 10.1590/0034-7167-2025-0220pt

**Authors:** Fernanda Cirne Lima Weston, Aline Branco, Isabela Ouriques Borghetti, Irani Iracema de Lima Argimon

**Affiliations:** I Pontifícia Universidade Católica do Rio Grande do Sul. Porto Alegre, Rio Grande do Sul, Brasil; II Grupo Hospitalar Conceição. Porto Alegre, Rio Grande do Sul, Brasil

**Keywords:** Cuidados Paliativos, Cuidados de Fim de Vida, Cuidadores, Sobrecarga do Cuidador, Morte.

## Abstract

**Objetivos:**

identificar barreiras e facilitadores percebidos por cuidadores informais que impactam a assistência a adultos e idosos em fim de vida no Brasil.

**Métodos:**

revisão sistemática de estudos qualitativos, seguindo o modelo JBI, com análise por meta-agregação.

**Resultados:**

incluíram-se 13 estudos. Foram identificados cinco achados sintetizados. Os cuidadores relataram como positivos para o cuidado de fim de vida o acesso a uma equipe multidisciplinar capacitada e afetuosa, a presença de uma rede de apoio informal e, principalmente, familiar, uma comunicação clara que aborde a temática do fim da vida, o desenvolvimento da resiliência, e o apoio em crenças e religiões. Como barreiras, foram identificados a falta de preparo dos profissionais e do ambiente hospitalar para o atendimento a esse público, e sentimentos de sobrecarga física, psicológica e financeira.

**Considerações Finais:**

os resultados encontrados têm implicações práticas, permitindo que sejam pensadas estratégias para mitigar as dificuldades enfrentadas pelos cuidadores no contexto brasileiro.

## INTRODUÇÃO

A população brasileira cresceu cerca de 7,6% entre os anos de 2012 e 2021, e o número de pessoas idosas apresentou um crescimento superior ao de todas as outras faixas etárias no período estudado^(1)^. Essa alteração demográfica, caracterizada pelo aumento na expectativa de vida, tem como consequência a maior prevalência de doenças crônicas não transmissíveis, que exigem um serviço de saúde estruturado para atendê-las^(2)^.

Dentro dessa categoria, pacientes adultos e idosos em fim de vida, ou seja, que têm expectativa de vida de meses ou menos, exigem cuidados constantes, já que apresentam intensificação de sintomas como dor e fadiga^(3-5)^. Esses pacientes são elegíveis para os cuidados paliativos, apesar de esses serviços não serem exclusivos às pessoas em fim de vida, e podem manifestar ainda nível de consciência reduzido, dificuldade na deambulação, entre outras barreiras para o autocuidado, tornando essencial a presença de um cuidador informal^(5)^.

Cuidadores informais não costumam ter qualificação profissional, prestando seus cuidados por princípios relacionais. Portanto, incluem-se familiares, amigos ou outras pessoas que não recebem remuneração para o exercício do cuidado. Entre as motivações para prestá-lo, incluem-se valores culturais e normas sociais, desde crenças religiosas, como a de que a pessoa está inserida em um papel de cuidador por vontade de Deus, até a percepção de que o serviço de saúde é insuficiente, o que exigiria a presença constante de um familiar para que não fossem cometidos erros^(6)^.

Os cuidadores informais assumem tarefas como a alimentação do paciente, administração de medicamentos, além de acompanhamento durante hospitalizações, apoiando-o emocionalmente^(7)^. Essas atividades, além de representarem grande sobrecarga física e emocional, tornam-nos uma presença constante e cotidiana na vida do paciente, permitindo que identifiquem as barreiras e facilitadores para o cuidado integral da pessoa em fim de vida. Conceitua-se “barreira” como os fatores que impedem e/ou dificultam que as necessidades físicas, psicológicas, sociais e espirituais da pessoa em fim de vida sejam atendidas. Em contrapartida, fatores “facilitadores” são os que permitem ou estimulam o atendimento das necessidades integrais desse paciente^(8)^.

Pesquisas internacionais demonstram haver múltiplas barreiras e facilitadores que impactam a utilização dos serviços de cuidados paliativos. O desconhecimento e medo dos cuidados paliativos, tanto por parte dos profissionais quanto dos cuidadores e dos pacientes, a dificuldade no acesso em áreas rurais, e a falta de comunicação entre médicos e equipe multidisciplinar são exemplos de fatores que dificultam o acesso a esses serviços, podendo ser também identificados pelos cuidadores informais da pessoa em fim de vida^(9,10)^.

Os obstáculos para o cuidado do paciente em fim de vida variam entre os países devido às diferentes estruturas de serviços de saúde, cultura e religião predominante da população^(10)^. Portanto, a presente revisão é construída com foco em estudos desenvolvidos nacionalmente a fim de respeitar essas peculiaridades.

Uma busca preliminar foi realizada na base de registros PROSPERO e na base de dados LILACS, em dezembro de 2023, para verificar se havia uma revisão sistemática que buscasse mapear quais as barreiras e facilitadores identificados por cuidadores informais no cuidado do paciente em fim de vida no Brasil. Entretanto, apesar de haver pesquisas brasileiras que abordassem as percepções e experiências dos cuidadores, não foram observadas revisões que buscassem reuni-las e sistematizá-las^(7,11)^. Considerando que a percepção desses cuidadores pode ser utilizada como recurso na qualificação do cuidado no fim da vida e dos serviços de saúde, torna-se necessária conhecê-las e sistematizá-las.

## OBJETIVOS

Identificar as barreiras e facilitadores percebidas e vivenciadas pelos cuidadores informais no cuidado de adultos e idosos em fim de vida no Brasil.

## MÉTODOS

### Aspectos éticos

Por se tratar de revisão sistemática, não foi necessária a aprovação pelo Comitê de Ética em Pesquisa.

### Delineamento da pesquisa

Optou-se por elaborar revisão sistemática de estudos qualitativos, seguindo-se a metodologia proposta pelo JBI^(12)^. Estudos qualitativos permitem, por meio da perspectiva da pessoa, identificar fragilidades e forças de intervenções em saúde, compreendendo o porquê de sua aplicabilidade, ou não, em cada contexto. O protocolo do estudo foi registrado (CRD42024523211) na plataforma PROSPERO.

### Identificação da questão de pesquisa

A questão de pesquisa “Quais as barreiras e facilitadores percebidos e vivenciados por cuidadores informais no cuidado dos pacientes adultos e idosos em fim de vida no Brasil?” foi elaborada por meio do acrônimo PICo (População, Fenômeno de Interesse e Contexto) a saber: P - cuidadores informais de pacientes; I - barreiras e facilitadores no cuidado em saúde; e Co - cuidados de fim de vida^(12)^. Como critérios de inclusão, os materiais selecionados deveriam ser estudos qualitativos primários, incluindo abordagens como estudos de caso e etnografia, realizados no Brasil, disponíveis *online* na íntegra e tendo como ênfase a percepção dos cuidadores informais que prestam ou tenham prestado assistência a um paciente em fim de vida. Artigos que também abordassem a percepção de profissionais de saúde poderiam ser incluídos, desde que fosse possível identificar se os entrevistados eram cuidadores informais ou formais, permitindo que fossem analisadas, para este estudo, somente as falas dos cuidadores informais.

Foram excluídos estudos que abordassem cuidadores de pacientes pediátricos (<18 anos) e materiais que não explicitassem a expectativa de vida do paciente ou que não o definissem como paciente em fim de vida. Estudos que utilizavam somente termos como “prognóstico negativo” e “em cuidados paliativos”, sem explicitar a expectativa de vida ou definir o paciente como em fim de vida e/ou com doença terminal (utilizados no presente artigo como sinônimos^(3)^), não foram incluídos na seleção. No caso de serem encontrados tanto artigos derivados de tese/dissertação quanto de tese/dissertação original, optou-se por selecionar os artigos publicados para revisão.

### Estratégia de busca

A busca foi realizada no dia 15 de março de 2024, abrangendo bases de dados de literatura publicada e cinzenta^(12)^, conforme recomendado pelo JBI. As buscas nas bases de dados foram realizadas a partir de descritores do *Medical Subject Headings*, e a estratégia de busca foi elaborada com o apoio de uma bibliotecária especialista na área da saúde (Quadro 1). Aplicaram-se os descritores nas bases de dados Scopus, PubMed, LILACS, SciELO e *Web of Science*, e para localizar literatura cinzenta, também foram utilizadas as bases SciELO *Preprints* e Catálogo de Teses e Dissertações da Coordenação de Aperfeiçoamento de Pessoal de Nível Superior (CAPES).

**Quadro 1:** Estratégias de busca utilizadas nas bases de dados, Porto Alegre, Rio Grande do Sul, Brasil, 2024

Fonte de informação	Estratégia de busca
LILACS	*(“Terminally Ill” OR “Ill, Terminally” OR “end of life care” OR “End of Life Care” OR “End-Of-Life Care” OR “Care, End-Of-Life” OR “End-Of-Life Cares” OR “Terminal Care” OR “Care, Terminal”) AND (caregivers OR caregiver OR carers OR carer OR “Care Givers” OR “Care Giver” OR “Spouse Caregivers” OR “Caregiver, Spouse” OR “Caregivers, Spouse” OR “Spouse Caregiver” OR “Family Caregivers” OR “Caregiver, Family” OR “Caregivers, Family” OR “Family Caregiver” OR “Informal Caregivers” OR “Caregiver, Informal” OR “Caregivers, Informal” OR “Informal Caregiver”) AND ( db:(“LILACS”))*
PubMed	*(“Terminally Ill”[MeSH Terms] OR “ill terminally”[All Fields] OR “Terminal Care”[MeSH Terms] OR “end of life care”[All Fields] OR “end of life care”[All Fields] OR “end of life care”[All Fields] OR “care end of life”[All Fields] OR “End-Of-Life Cares”[All Fields] OR “care terminal”[All Fields]) AND (“caregivers”[MeSH Terms] OR (“caregiver s”[All Fields] OR “caregivers”[MeSH Terms] OR “caregivers”[All Fields] OR “caregiver”[All Fields] OR “caregiving”[All Fields]) OR (“caregivers”[MeSH Terms] OR “caregivers”[All Fields] OR “carer”[All Fields] OR “carers”[All Fields] OR “carer s”[All Fields]) OR (“caregivers”[MeSH Terms] OR “caregivers”[All Fields] OR “carer”[All Fields] OR “carers”[All Fields] OR “carer s”[All Fields]) OR “Care Givers”[All Fields] OR “Care Giver”[All Fields] OR “Spouse Caregivers”[All Fields] OR “caregiver spouse”[All Fields] OR “caregivers spouse”[All Fields] OR “Spouse Caregiver”[All Fields] OR “Family Caregivers”[All Fields] OR “caregiver family”[All Fields] OR “caregivers family”[All Fields] OR “Family Caregiver”[All Fields] OR “Informal Caregivers”[All Fields] OR “caregiver informal”[All Fields] OR “caregivers informal”[All Fields] OR “Informal Caregiver”[All Fields]) AND (“brasil”[All Fields] OR (“brazil”[MeSH Terms] OR “brazil”[All Fields] OR “brazil s”[All Fields] OR “brazils”[All Fields]) OR (“brazilian people”[Supplementary Concept] OR “brazilian people”[All Fields] OR “brazilians”[All Fields] OR “brazilian”[All Fields]) OR (“brazilian people”[Supplementary Concept] OR “brazilian people”[All Fields] OR “brazilians”[All Fields] OR “brazilian”[All Fields]))*
Scopus	*TITLE-ABS-KEY ( ( “Terminally Ill” OR “Ill, Terminally” OR “end of life care” OR “End of Life Care” OR “End-Of-Life Care” OR “Care, End-Of-Life” OR “End-Of-Life Cares” OR “Terminal Care” OR “Care, Terminal” ) AND ( caregivers OR caregiver OR carers OR carer OR “Care Givers” OR “Care Giver” OR “Spouse Caregivers” OR “Caregiver, Spouse” OR “Caregivers, Spouse” OR “Spouse Caregiver” OR “Family Caregivers” OR “Caregiver, Family” OR “Caregivers, Family” OR “Family Caregiver” OR “Informal Caregivers” OR “Caregiver, Informal” OR “Caregivers, Informal” OR “Informal Caregiver” ) AND ( brasil OR brazil OR brazilian or brazilians ) )*
*Web of Science*	*(“Terminally Ill” OR “Ill, Terminally” OR “end of life care” OR “End of Life Care” OR “End-Of-Life Care” OR “Care, End-Of-Life” OR “End-Of-Life Cares” OR “Terminal Care” OR “Care, Terminal”) AND (Caregivers OR Caregiver OR Carers OR Carer OR “Care Givers” OR “Care Giver” OR “Spouse Caregivers” OR “Caregiver, Spouse” OR “Caregivers, Spouse” OR “Spouse Caregiver” OR “Family Caregivers” OR “Caregiver, Family” OR “Caregivers, Family” OR “Family Caregiver” OR “Informal Caregivers” OR “Caregiver, Informal” OR “Caregivers, Informal” OR “Informal Caregiver”) AND (Brasil OR Brazil OR brazilian OR brazilians) (All Fields)*
SciELO *Preprints*	*(“Terminally Ill” OR “Ill, Terminally” OR “end of life care” OR “End of Life Care” OR “End-Of-Life Care” OR “Care, End-Of-Life” OR “End-Of-Life Cares” OR “Terminal Care” OR “Care, Terminal”) AND (Caregivers OR Caregiver OR Carers OR Carer OR “Care Givers” OR “Care Giver” OR “Spouse Caregivers” OR “Caregiver, Spouse” OR “Caregivers, Spouse” OR “Spouse Caregiver” OR “Family Caregivers” OR “Caregiver, Family” OR “Caregivers, Family” OR “Family Caregiver” OR “Informal Caregivers” OR “Caregiver, Informal” OR “Caregivers, Informal” OR “Informal Caregiver”)*
SciELO (Português)	(“Doente Terminal” *OR* “Paciente Terminal” *OR* “Assistência Terminal” *OR* “Cuidados de Fim de Vida”) *AND* (Cuidador *OR* “Cuidador Familiar” *OR* “Cuidador de Família” *OR* “Cuidadores Cônjuges” *OR* “Cuidadores Familiares” *OR* “Cuidadores Informais” *OR* “Cuidadores de Família” *OR* “Cônjuges Cuidadores” *OR* “Familiar Cuidador” *OR* “Familiares Cuidadores” *OR* “Outro Apoiador”)
SciELO (Inglês)	*(“Terminally Ill” OR “Ill, Terminally” OR “end of life care” OR “End of Life Care” OR “End-Of-Life Care” OR “Care, End-Of-Life” OR “End-Of-Life Cares” OR “Terminal Care” OR “Care, Terminal”) AND (caregivers OR caregiver OR carers OR carer OR “Care Givers” OR “Care Giver” OR “Spouse Caregivers” OR “Caregiver, Spouse” OR “Caregivers, Spouse” OR “Spouse Caregiver” OR “Family Caregivers” OR “Caregiver, Family” OR “Caregivers, Family” OR “Family Caregiver” OR “Informal Caregivers” OR “Caregiver, Informal” OR “Caregivers, Informal” OR “Informal Caregiver”)*
Teses e Dissertações da Coordenação de Aperfeiçoamento de Pessoal de Nível Superior	Terminal *AND* Cuidadores

Realizou-se uma busca inicial na base PubMed com o auxílio da bibliotecária para verificar se a estratégia estava adequada à pergunta de pesquisa proposta. Após a verificação dos resultados iniciais, prosseguiu-se com a aplicação da estratégia de busca em todas as bases. As buscas foram realizadas por meio do acesso institucional Comunidade Acadêmica Federada da CAPES. Não foram aplicados filtros de idioma, assim como não foi feita uma restrição do ano de publicação.

### Análise dos dados

Os materiais encontrados nas bases de dados foram incluídos no aplicativo *Rayyan* ^®^ para maior organização e detecção de duplicatas. A triagem dos estudos é apresentada por meio do *Preferred Reporting Items for Systematic Reviews and Meta-Analyses* (PRISMA)^(13)^. O processo de busca foi realizado por dois pesquisadores, que iniciaram com a leitura dos títulos e resumos dos artigos retornados e, posteriormente, leram os artigos na íntegra. Durante a triagem dos estudos, acionava-se um terceiro pesquisador em casos de discrepâncias nas decisões que não fossem resolvidas após discussão entre os dois pesquisadores iniciais.

Após a triagem dos estudos pelo *Rayyan* ^®^, os artigos foram inseridos no *software* JBI SUMARI^®^ para aplicação do instrumento de avaliação crítica *Checklist for Qualitative Research*, preenchido por duas pesquisadoras para posterior comparação das avaliações. Somente foram selecionados para revisão os estudos que, além de seguirem os critérios de inclusão já citados, cumpriam com os critérios 2, 4, 8 e 10 do *checklist*.

O mapeamento dos dados ocorreu por meio da *JBI Qualitative data extraction tool*, instrumento que foi preenchido de forma independente por duas pesquisadoras e, posteriormente, tiveram seus resultados comparados. A análise de dados foi realizada por meio da meta-agregação, por permitir a identificação de padrões em diferentes entrevistas e também por já direcionar os resultados para a prática clínica^(12)^.

Seguindo-se a meta-agregação, a primeira etapa de análise dos estudos consistiu em lê-los na íntegra para identificar os achados (*findings*), que são extrações literais das palavras do autor do texto. Todos os achados devem ser acompanhados de uma ilustração, que é a transcrição literal da fala do entrevistado. Após reunir os achados às ilustrações, as pesquisadoras eram responsáveis por atribuir um nível de credibilidade à junção. Este é dividido em inequívoco (*unequivocal*, no qual a ilustração é claramente associada aos achados), credível (*credible,* no qual a ilustração tem uma associação aos achados, porém não tão clara) e não confirmado (*unsupported,* no qual a ilustração não oferece suporte à interpretação do autor). Todos os achados não confirmados não foram considerados para a análise dos resultados.

Após ser atribuído o nível de credibilidade, passou-se para a segunda etapa da meta-agregação, criando-se categorias a partir da reunião de achados que tivessem um significado similar. Por fim, as categorias que compartilhassem significados similares também foram reunidas nos chamados achados sintetizados (*synthesized findings*). Os achados sintetizados foram considerados de alta confiabilidade quando compostos somente por categorias inequívocas; de moderada confiabilidade, quando compostos por categorias inequívocas e credíveis; e de baixa confiabilidade, quando compostos somente por categorias credíveis.

## RESULTADOS

Após a aplicação das estratégias de busca nas bases de dados, identificou-se um total de 315 publicações, conforme fluxograma PRISMA (Figura 1). Removendo-se as duplicatas, mantiveram-se 216. Na leitura do título e resumo, 160 foram excluídas, e somente em um conflito, a terceira pesquisadora foi acionada. Foram selecionados 56 materiais para leitura na íntegra. Destes 56, 15 foram selecionados pela leitura e, após a aplicação do *Checklist for Qualitative Research*, 13 foram selecionados para a revisão sistemática.

**Figura 1:** Fluxograma *Preferred Reporting Items for Systematic Reviews and Meta-Analyses*, Porto Alegre, Rio Grande do Sul, Brasil, 2024

Dos materiais selecionados, nove (69,2%) foram realizados em serviços de internação domiciliar^(14-22)^ e cinco (38,5%), em ambiente hospitalar^(7,11,21,23,24)^. Um dos artigos foi realizado em ambas as modalidades^(20)^. Entre todos os estudos, oito (61,5%) foram feitos na região Sul, três (23%), na região Sudeste, e dois (15,4%), na região Nordeste (Quadro 2).

**Quadro 2:** Características dos estudos selecionados, Porto Alegre, Rio Grande do Sul, Brasil, 2024

Autores (Ano)	Amostra | Diagnóstico dos pacientes	Local (região)	Instrumento de coleta
Oliveira *et al.* (2011)^(14)^	11 cuidadores familiares | Câncer	Serviço de internação domiciliar vinculado a um hospital universitário (Sul)	Entrevistas narrativas
Oliveira *et al.* (2012)^(15)^	11 cuidadores familiares | Câncer	Serviço de internação domiciliar vinculado a um hospital universitário (Sul)	Entrevistas narrativas
Vasques *et al.* (2017)^(23)^	24 cuidadores familiares | Não identificado	Unidade de clínica médica (Sul)	Observação e entrevista
Oliveira *et al.* (2013)^(16)^	11 cuidadores familiares | Câncer	Serviço de internação domiciliar vinculado a um hospital universitário (Sul)	Entrevistas narrativas
Barbosa *et al.* (2020)^(7)^	15 cuidadores familiares | Câncer	Hospital filantrópico (Nordeste)	Entrevistas semiestruturadas
Nietsche *et al.* (2013)^(17)^	Duas enfermeiras, uma auxiliar de enfermagem, uma médica, uma nutricionista e quatro cuidadores familiares | Não identificado	Serviço de internação domiciliar vinculado a um hospital universitário (Sul)	Entrevistas semiestruturadas
Cogo *et al.* (2016)^(18)^	Oito enfermeiros, sete médicos e sete cuidadores familiares | Não identificado	Serviço de internação domiciliar vinculado a um hospital universitário (Sul)	Entrevista semiestruturada
Oliveira *et al.* (2012)^(19)^	11 cuidadores familiares | Câncer	Serviço de internação domiciliar vinculado a um hospital universitário (Sul)	Entrevistas narrativas
Souza & Turrini (2011)^(24)^	Sete cuidadores familiares | Câncer	Serviço ambulatorial de um hospital universitário público (Sudeste)	Entrevistas não diretivas
Santos (2019)^(21)^	18 cuidadores familiares | Câncer	Serviço de atendimento domiciliar vinculado a uma unidade de cuidados paliativos de um hospital filantrópico (Nordeste)	Entrevista aberta
Benites AC *et al.* (2022)^(11)^	Dez cuidadores familiares | Câncer	Hospital e centro oncológico (Sudeste)	Entrevista fenomenológica
Santos MJ (2009)^(20)^	Dez cuidadores familiares, três enfermeiras, quatro técnicos de enfermagem e um auxiliar de enfermagem | Câncer	Serviço de cuidados paliativos domiciliar, ambulatorial e internação hospitalar (Sul)	Entrevista semiestruturada
Fonseca COS (2012)^(22)^	Sete cuidadores familiares | Câncer	Serviço de internação domiciliar (Sudeste)	Entrevista semidirigida

Em relação aos diagnósticos estudados, dez (76,9%) estudos abordaram cuidadores de pacientes com câncer, apesar de não especificarem o tipo, e três (23,1%) não identificaram o diagnóstico. Após a realização da meta-agregação, foram identificados cinco achados sintetizados, expostos abaixo juntamente com as categorias que os compõem (Quadro 3).

**Quadro 3:** Achados sintetizados e categorias identificadas por meio da meta-agregação, Porto Alegre, Rio Grande do Sul, Brasil, 2024

Achados sintetizados	Categorias
1. Os cuidadores relatam que o fácil acesso a uma equipe multidisciplinar capacitada e afetuosa, que respeita a autonomia do paciente em fim de vida, é central para a realização de um bom cuidado	Acesso à equipe multidisciplinar
	Equipe multidisciplinar afetuosa e respeitosa
	Cuidado e tarefas compartilhados com profissionais
	Apoio da equipe de enfermagem
	Cuidado domiciliar
	Dificuldade em identificar e atender às necessidades do paciente
2. Os cuidadores afirmam que é fundamental ter uma rede de apoio informal para o compartilhamento do cuidado, evitando sobrecarga física, psicológica e financeira	Apoio familiar
	Sobrecarga física e emocional
	Ausência de apoio familiar
	Solidão
	Dificuldade financeira
	Criação de uma rede de apoio
3. Os cuidadores percebem a importância de uma comunicação clara e efetiva que aborde a temática da morte e o morrer entre a equipe multidisciplinar, o cuidador e o paciente	Comunicação inadequada entre o cuidador, o paciente e a equipe multidisciplinar
	Comunicação que permita falar sobre a morte
	Comunicação adequada com e entre a equipe multidisciplinar
4. Os cuidadores percebem uma falta de preparo dos profissionais e do ambiente hospitalar para o atendimento do paciente em fim de vida, assim como desconfiam dos serviços de cuidados paliativos	Afastamento da equipe multidisciplinar
	Limitações das diretivas antecipadas de vontade
	Espaço hospitalar
	Desconfiança dos cuidados paliativos e demais serviços de saúde
5. Os cuidadores descrevem diferentes ferramentas, como desenvolvimento da resiliência e apoio em crenças e religiões, para aceitação da terminalidade e enfrentamento do papel de cuidador	Resiliência
	Crenças e religião
	Aceitação da morte

### Achado sintetizado 1: Os cuidadores relatam que o fácil acesso a uma equipe multidisciplinar capacitada e afetuosa, que respeita a autonomia do paciente em fim de vida, é central para a realização de um bom cuidado

Este achado sintetizado foi criado a partir de seis categorias, e é considerado de moderada confiabilidade, por abranger 24 achados inequívocos e quatro achados credíveis. Identifica-se, de acordo com as percepções dos cuidadores, que a preferência pelo ambiente do cuidado, sendo domiciliar ou hospitalar, se dá principalmente pelo fácil acesso à equipe multidisciplinar.

A categoria denominada “Acesso à equipe multidisciplinar” é formada por quatro achados. Alguns cuidadores relatam que preferem o ambiente hospitalar, por haver a presença constante dos profissionais de saúde. Em relação ao serviço domiciliar, sentem-se seguros ao terem a presença e o contato telefônico da equipe e saberem que podem entrar em contato 24 horas para dúvidas e apoio. Relatam que já conhecer previamente algum dos membros do serviço domiciliar os deixa menos receosos de acessá-lo.

A categoria “Equipe multidisciplinar afetuosa e respeitosa” é formada por oito achados, realçando a importância de ter acesso a uma equipe multidisciplinar que demonstra paciência, afeto, respeito e disponibilidade tanto para o paciente quanto para o cuidador. A escuta ativa e o respeito às necessidades e desejos do paciente são valorizados pelos cuidadores.

Em relação à categoria “Cuidado e tarefas compartilhados com profissionais”, seis achados a compõem, sendo que os cuidadores se sentem mais seguros e com menor sobrecarga quando o cuidado e as tarefas diárias são compartilhados com algum profissional, que pode ser tanto um membro da equipe de saúde quanto um empregado doméstico, responsável pela limpeza e organização da casa. Citam as orientações recebidas pela equipe de saúde do serviço domiciliar, assim como a divisão de tarefas, como um revezamento na realização de curativos entre os cuidadores formais e informais.

A categoria “Apoio da equipe de enfermagem” é composta de três achados. Os cuidadores valorizam o amparo e conforto proporcionados especificamente pela equipe de enfermagem, percebendo-os como amparadores tanto no meio domiciliar quanto no ambiente hospitalar. Enquanto isso, a categoria “Cuidado domiciliar” é composta por três achados. Os cuidadores percebem que os serviços de atendimento domiciliar facilitam o acesso aos recursos hospitalares, como equipamentos médicos e atendimentos de urgência. Também reconhecem que essa modalidade permite maior exercício da autonomia por parte do paciente.

Por fim, a categoria “Dificuldade em identificar e atender às necessidades do paciente” é formada por quatro achados, demonstrando as dificuldades relatadas pelos cuidadores em identificar e manejar as necessidades de saúde do paciente. Questões como dor não controlada e a maior irritabilidade do paciente impactam a percepção de capacidade dos cuidadores, tornando-os receosos por não estarem prestando um cuidado adequado.

### Achado sintetizado 2: Os cuidadores afirmam que é fundamental ter uma rede de apoio informal para o compartilhamento do cuidado, evitando sobrecarga física, psicológica e financeira

Criado a partir de seis categorias, este achado sintetizado é considerado de moderada confiabilidade, abrangendo 28 achados, sendo dois moderados e 26 inequívocos. Enfatiza a importância de uma rede de apoio comunitária e, principalmente, familiar, tanto no compartilhamento do cuidado quanto no apoio financeiro. A possibilidade de um rodízio intrafamiliar reduziria o sentimento de sobrecarga e a solidão do cuidador principal que, por vezes, teme ser o único responsabilizado caso haja a morte do paciente.

A categoria “Apoio familiar” é composta por três achados, realçando a importância de o cuidador ter acesso a uma rede de apoio familiar para o rodízio no cuidado do paciente em fim de vida. Cita a preferência pelo serviço domiciliar quando há ajuda de demais membros da família, e valorizam o apoio emocional que recebem dos familiares. Enquanto isso, a categoria “Sobrecarga física e emocional” é formada por dez achados. Há diferentes sentimentos e sintomas físicos que emergem do cuidado do paciente em fim de vida e dificultam a sua realização, incluindo medo, ansiedade, angústia, depressão, cansaço, estresse, desespero, desânimo e falta de reconhecimento. Destaca-se que ser mãe cuidando de um filho em fim de vida dificulta ainda mais a elaboração do luto, aumentando a sobrecarga emocional.

A categoria “Ausência de apoio familiar”, constituída por cinco achados, demonstra as experiências de cuidadores quando há falta de apoio familiar, incluindo a falta de apoio financeiro e conflitos entre membros familiares. Percebe-se um viés de gênero nessas vivências, e os cuidadores costumam ser mulheres que não recebem apoio de demais membros homens da família. Há receio de ser atendido pelo serviço domiciliar, pois o cuidador pode ser responsabilizado por outros membros da família caso o paciente morra em domicílio.

Enquanto isso, a categoria “Solidão” é composta por três achados. Refere-se ao sentimento dos cuidadores de estarem desacompanhados e isolados no cuidado do paciente em fim de vida, incluindo ao receberem o diagnóstico inicial da doença. Percebe-se ainda a “Dificuldade financeira”, formada por cinco achados, demonstrando as limitações financeiras que impactam o cuidado, o acesso e o transporte aos serviços de saúde do paciente em fim de vida. Os cuidadores relatam conflito com os seus empregadores tanto durante internações hospitalares quanto na internação domiciliar, devido ao tempo despendido ao paciente, culminando em sua demissão.

Por fim, a categoria “Criação de uma rede de apoio” é composta por dois achados. Expõe a importância dada pelos cuidadores ao diálogo e ao vínculo estabelecido com demais pacientes e cuidadores que também estão em internação hospitalar em situações similares.

### Achado sintetizado 3: Os cuidadores percebem a importância de uma comunicação clara e efetiva que aborde a temática da morte e o morrer entre a equipe multidisciplinar, o cuidador e o paciente

Este achado sintetizado é composto por três categorias, e tem alta confiabilidade, sendo composto por oito achados inequívocos. Há no relato dos cuidadores o reconhecimento da importância da comunicação entre a tríade formada por paciente, cuidador principal e equipe assistente. Também afirma maior segurança e confiança quando percebem uma comunicação integrada entre diferentes membros da equipe multidisciplinar. Constata que diálogos sobre a morte e o processo de morrer são difíceis, porém necessários.

A categoria “Comunicação inadequada entre o cuidador, o paciente e a equipe multidisciplinar”, formada por quatro achados, expõe que o uso de linguagem técnico-científica e de explicações vagas para explicar o prognóstico negativo por parte dos profissionais impacta negativamente o cuidado do paciente em fim de vida. A falta de comunicação tanto por parte da equipe quanto por parte dos cuidadores sobre o fim da vida também é reconhecida como uma barreira para a assistência. A categoria “Comunicação que permita falar sobre a morte”, constituída por dois achados, demonstra a importância de se estabelecer uma comunicação com a equipe multidisciplinar e com o paciente que permita discutir sobre a morte, possibilitando o compartilhamento de informações corretas entre cuidador, paciente e familiares.

Em contraponto, a categoria “Comunicação adequada com e entre a equipe multidisciplinar”, formada por dois achados, refere-se à importância do estabelecimento de uma comunicação efetiva e esclarecedora entre os membros da equipe multidisciplinar, bem como entre estes e os cuidadores. Ressalta-se também a necessidade de integração entre a equipe, de forma que todos repassem a mesma informação ao cuidador.

### Achado sintetizado 4: Os cuidadores percebem uma falta de preparo dos profissionais e do ambiente hospitalar para o atendimento do paciente em fim de vida, assim como desconfiam dos serviços de cuidados paliativos

Criado a partir de quatro categorias, este achado sintetizado aborda questões relativas ao afastamento da equipe multidisciplinar em relação ao paciente em fim de vida, às limitações percebidas pelos cuidadores sobre a aplicação das diretivas antecipadas de vontade (DAVs), às características do espaço hospitalar e à desconfiança ou desconhecimento acerca dos cuidados paliativos e demais serviços de saúde. É considerado de moderada credibilidade, sendo constituído por duas categorias credíveis e oito inequívocas.

A categoria “Afastamento da equipe multidisciplinar” é composta por dois achados, tratando sobre a percepção dos cuidadores de que a equipe multidisciplinar evita se aproximar do paciente em fim de vida. Há a constatação dos familiares que, ao haver o estabelecimento do prognóstico negativo, a assistência dos profissionais de saúde ao paciente diminui. A categoria “Limitações das diretivas antecipadas de vontade”, constituída por três achados, expõe ainda a falta de preparo profissional e a falta de respaldo legal, que impactam a concretização das DAVs. Os cuidadores têm receios ligados às possíveis implicações legais derivadas das DAVs, e demonstram ainda ter uma esperança de cura do paciente em fim de vida, limitando a concretização das DAVs.

As percepções dos cuidadores sobre o “Espaço hospitalar” também demonstram as características do ambiente hospitalar que impactam o cuidado do paciente em fim de vida. Destacam os aspectos negativos desse espaço, realçando que, por conta dele, fica mais difícil o exercício da autonomia, e há maiores chances de o paciente contrair uma infecção, esta última principalmente pelos quartos serem compartilhados com mais pessoas.

Por fim, a categoria “Desconfiança dos cuidados paliativos e demais serviços de saúde” é composta por três achados. Os cuidadores informais percebem que a sua desconfiança dos serviços de saúde e o desconhecimento do objetivo dos cuidados paliativos impactam o acesso a estes. Desconfiam da qualidade da assistência do serviço por ser oferecido pelo Sistema Único de Saúde. Em relação aos cuidados paliativos, têm uma ideia prévia de que são um serviço que as pessoas ingressam somente para morrer. Os cuidadores também relatam sentir medo de contratar alguém para auxiliar no cuidado do paciente em fim de vida por temerem que seu familiar seja maltratado.

### Achado sintetizado 5: Os cuidadores descrevem diferentes ferramentas, como desenvolvimento da resiliência e apoio em crenças e religiões, para aceitação da terminalidade e enfrentamento do papel de cuidador

Este achado sintetizado é composto por três categorias, e tem confiabilidade moderada por apresentar sete categorias inequívocas e uma credível. Refere-se ao desenvolvimento da resiliência, ao papel das crenças e da religião enquanto apoio aos cuidadores e à aceitação da morte.

Em relação à “Resiliência”, constituída de quatro achados, expõem-se sentimentos adaptativos que emergem em situações de risco e de adversidade. Trata-se do surgimento da coragem e de sentimentos de amor nos cuidadores, os quais parecem fortalecê-los diante desse desafio que é o cuidar. Aborda a existência de sentimentos relacionados à gratidão e do mecanismo do humor, por meio do “ser palhaça”.

A categoria “Crenças e religião”, formada por três achados, discorre sobre as crenças e religião como fonte de apoio para o cuidador. Expõe o valor da fé e da religiosidade no processo de cuidar. Os cuidadores relatam que a religião, a fé e a crença em vida após a morte parecem facilitar o processo de aceitação da morte, bem como ajudam a entender o sofrimento vivenciado.

Por fim, a categoria “Aceitação da morte” é formada por três achados, apresentando o reconhecimento e a aceitação da finitude do paciente em fim de vida por parte do cuidador. Versa sobre a aceitação da terminalidade da vida e sobre o poder se preparar previamente para os momentos seguintes à morte do paciente, como na preparação dos ritos funerários. Também aborda a aceitação da morte por meio da visão de que ela representa o fim do sofrimento.

## DISCUSSÃO

Os achados dos estudos suscitam algumas reflexões sobre as diferentes barreiras e facilitadores identificados pelos cuidadores informais na assistência ao paciente em fim de vida no Brasil. Relatam que o fácil acesso a uma equipe multidisciplinar capacitada e afetuosa é central para a realização de um bom cuidado tanto para os serviços de internação domiciliar quanto para o meio hospitalar.

É necessário considerar esta informação na operacionalização de serviços para o atendimento em final de vida no Brasil, compreendendo a importância que tem para os cuidadores a presença e o contato constante dos profissionais de saúde para suporte e orientações. A literatura demonstra, principalmente, a relevância de serem ofertados números telefônicos que atendam após horário comercial para acolher preocupações referentes ao agravamento de sintomas físicos e psicológicos comuns na etapa final de vida^(25,26)^.

Reconhece-se que a limitação de recursos financeiros e humanos, comum em diferentes regiões e instituições no Brasil, pode dificultar ou impedir a concretização desses serviços^(24)^. Contudo, é importante ressaltar os impactos positivos derivados de ter o acesso constante a uma equipe multidisciplinar que, além de reduzir sintomas do paciente, ainda reduz o sentimento de sobrecarga e melhora o bem-estar e o humor do cuidador^(27)^.

Além da importância de compartilhar o cuidado com uma equipe preparada tecnicamente, os cuidadores também valorizam profissionais que demonstram afeto e respeito tanto ao paciente quanto aos seus familiares. Diretrizes de diferentes países, como Austrália e Singapura, trazem a relevância da equipe multiprofissional apresentar uma comunicação honesta, sensível e que respeite os desejos e necessidades do paciente a fim de ser criado um vínculo de confiança^(28)^. Nas diretrizes nacionais do Canadá e Índia, também se registra como competência necessária para o cuidado da pessoa em fim de vida a comunicação empática e compassiva, corroborando os achados brasileiros^(29,30)^.

Além de poderem compartilhar o cuidado do paciente com a equipe multidisciplinar, os cuidadores trazem a necessidade de também terem uma rede de apoio informal formada, principalmente, por familiares. Outros estudos já demonstraram a importância de não designar tarefas de cuidado somente para o cuidador principal, mas compartilhá-las entre diversos membros da família^(31)^. Na presente revisão, os cuidadores percebem que o rodízio no cuidado evita a sobrecarga física e psicológica, assim como possibilita que o atendimento ocorra em serviço domiciliar, não hospitalar.

Ademais, cuidadores relatam a importância de receber apoio financeiro de outros membros da família para que possam acessar os serviços de saúde e manter o devido tratamento. Essa necessidade é também observada em pesquisas em outros países em desenvolvimento: em Uganda, assim como no Brasil, gastos com transporte público e alimentação sobrecarregam o cuidador principal, forçando-o a vender bens e a utilizar todas as suas poupanças^(32)^. Em ambas as nações, também há o relato de constantes conflitos com os empregadores, resultando em demissões e ainda maior limitação financeira.

Além da necessidade de apoio familiar, os cuidadores percebem a importância de uma comunicação clara e efetiva que aborde a temática da morte entre a equipe multidisciplinar, cuidador e paciente. Entende-se como comunicação efetiva aquela que ocorre de forma clara, direta, conjunta e rotineira, e a sua insuficiência se torna um dos principais fatores que contribuem para casos de eventos adversos e para a diminuição da qualidade de vida dos pacientes^(33)^.

Apesar de este ser o único achado sintetizado de alta confiabilidade, os profissionais de saúde afirmam não terem abertura para discutir questões que envolvam o fim da vida do paciente com seus familiares. Estudo realizado em Sergipe demonstra que as maiores dificuldades de implementação das DAVs são relativas à resistência familiar gerada por uma incompreensão do real prognóstico do paciente. Na mesma pesquisa, a equipe é cautelosa em relação às respostas emocionais dos familiares, havendo um movimento de evitação dos sentimentos de estresse, culpa, falha e impotência que podem surgir a depender da reação do cuidador^(34)^.

De encontro a essa constatação, estudo realizado na Inglaterra demonstra que pacientes terminais e seus familiares cuidadores possuem resistência em abordar a temática da morte e do morrer, trazendo esse assunto sempre de forma indireta^(35)^. Interessantemente, a autora também expõe que há a expectativa dos familiares de que quem traga essas questões seja sempre a equipe multidisciplinar e que tal abordagem seja direta, corroborando os achados da presente revisão.

Os cuidadores também percebem como barreira a falta de preparo dos profissionais e do hospital para acolhimento do paciente em fim de vida. Durante a internação, há um notável afastamento da equipe multidisciplinar quando é estabelecido o prognóstico negativo, o que também é identificado pelos próprios profissionais de saúde. Em diferentes estudos realizados no Brasil, enfermeiros e técnicos de enfermagem relatam que os colegas se distanciam da pessoa em cuidados de fim de vida e deixam de realizar procedimentos necessários para o conforto do paciente, atribuindo este comportamento à incapacidade do profissional de enfrentar o sofrimento e a morte^(36,37)^.

A proximidade com a morte também é um desafio para o cuidador informal, e este relata diferentes ferramentas para aceitação da terminalidade e exercício do seu papel. O desenvolvimento da resiliência, definida como a capacidade de se adaptar positivamente às situações adversas, é percebido como necessário para o enfrentamento da morte^(38)^. O bom-humor, a gratidão e o sentimento de amor surgem em outros estudos realizados com cuidadores de pacientes terminais e com demência, e são considerados forças psicológicas passíveis de serem estimuladas e fortalecidas para melhoria da qualidade de vida do cuidador^(39,40)^.

Além do desenvolvimento da resiliência, é frequente a busca por apoio em crenças e religião pelos cuidadores. De acordo com a Associação Europeia de Cuidados Paliativos, a espiritualidade é uma dimensão dinâmica da vida humana que está relacionada à forma como as pessoas se conectam a determinado momento de vida, a si próprio, aos outros e ao sagrado, e contribui para o desenvolvimento de esperança em relação ao futuro^(41)^. Enquanto isso, rituais religiosos auxiliam no processo de aceitação da morte e da perda, desenvolvendo a noção de encerramento de ciclo e facilitando a autoconsciência do cuidador informal em direção a uma maior apreciação dos eventos da vida.

Estudo realizado no Brasil demonstra a correlação entre a espiritualidade e a sobrecarga emocional de cuidadores familiares de pacientes sob cuidados paliativos exclusivos^(42)^. Os familiares que contam com uma espiritualidade elevada se sentem menos sobrecarregados, enquanto cuidadores com menores níveis de espiritualidade correm maior risco de sobrecarga emocional, ansiedade e estresse, abrindo-se um campo a ser acolhido e estimulado pelos profissionais de saúde.

### Limitações do estudo

A presente revisão possibilita a identificação das principais barreiras e facilitadores enfrentados pelos cuidadores informais durante o cuidado de pessoas em fim de vida no Brasil, mas deve ter suas limitações destacadas. Nenhum dos estudos selecionados foi realizado nas regiões Norte e Centro-Oeste, devendo-se questionar se os resultados podem ser generalizados para esses contextos. As pesquisas também foram realizadas majoritariamente com cuidadores de pacientes com câncer, questionando-se a aplicabilidade dos achados para outros diagnósticos como doenças neurodegenerativas, cardíacas, entre outras.

### Contribuições para a área da enfermagem

Apesar das limitações, os resultados encontrados têm implicações práticas e permitem que sejam pensadas estratégias para mitigar as dificuldades enfrentadas pelos cuidadores no contexto nacional. Abordagens institucionais como a manutenção de um telefone 24 horas para dúvidas dos cuidadores informais e o fortalecimento de uma cultura organizacional que preza pela saúde mental dos profissionais da assistência podem impactar positivamente o cuidado da pessoa em fim de vida. Capacitações para os profissionais de saúde, principalmente no que diz respeito à abordagem da temática da morte na sua comunicação com o paciente e o cuidador, também poderiam ser consideradas vantajosas.

O estímulo à resiliência e o respeito à espiritualidade, assim como o reforço desde o início do tratamento sobre a importância de haver um rodízio familiar nas tarefas, podem impactar a qualidade de vida dos cuidadores, tendo como consequência uma melhoria na assistência ao paciente. Portanto, as consultas de enfermagem com os cuidadores e os pacientes em fim de vida devem explicitamente orientar os cuidadores informais a organizar uma rede de apoio informal, visando compartilhar o cuidado e reduzir a sobrecarga física, psicológica e financeira.

Novos estudos podem ser realizados para avaliar a eficácia e aplicabilidade dessas intervenções, inclusive em ambientes com limitações de recursos, frequentes no contexto brasileiro. Também se identifica uma lacuna no conhecimento referente ao porquê do afastamento dos profissionais de saúde em relação à temática da morte, sendo necessárias pesquisas para compreender este fenômeno, tendo como objetivo evitá-lo e tornar o cuidado mais emocionalmente sustentável aos cuidadores formais.

## CONSIDERAÇÕES FINAIS

Diversos facilitadores e barreiras são relatados por cuidadores informais na assistência às pessoas em fim de vida no Brasil. O acesso a uma equipe multidisciplinar capacitada e afetuosa, a presença de uma rede de apoio informal e, principalmente, familiar, uma comunicação clara e efetiva que aborde a temática da terminalidade, o desenvolvimento da resiliência, e o apoio em crenças e religiões são fatores que os cuidadores percebem como positivos ao cuidado da pessoa em fim de vida.

Por outro lado, observam barreiras como a falta de preparo dos profissionais e do ambiente hospitalar para o atendimento a esse público. Também relatam sentimentos de sobrecarga física, psicológica e financeira, associando-os, principalmente, à falta de apoio familiar recebido.

A análise dessas barreiras e facilitadores permite que os profissionais de saúde elaborem estratégias de acolhimento mais adaptadas às vivências desses cuidadores. Por exemplo, durante uma consulta de enfermagem, uma prática valiosa seria apoiar e orientar o cuidador a identificar sua rede de apoio formal e informal, a fim de evitar a sobrecarga. A capacitação profissional em comunicação de más notícias também se mostra fundamental para uma abordagem eficaz sobre o fim da vida. Entretanto, reconhece-se que a melhoria dessas condições exigirá não apenas capacitação profissional, mas também políticas públicas eficazes e reorganizações institucionais que reconheçam e mitiguem a sobrecarga enfrentada pelos cuidadores informais no Brasil.

## Data Availability

Os dados de pesquisa estão disponíveis no corpo do artigo.
